# Experimental Investigation of the Dynamic Mechanical Properties of Polypropylene-Fiber-Reinforced Foamed Concrete at High Temperatures

**DOI:** 10.3390/polym15112544

**Published:** 2023-05-31

**Authors:** Longyang Chen, Penghui Li, Weiguo Guo, Ruifeng Wang, Dongjian Zhang, Meng Gao, Chang Peng

**Affiliations:** 1 School of Aeronautics, Northwestern Polytechnical University, Xi’an 710072, China; yang218cly@aliyun.com (L.C.); wang_rf1130@163.com (R.W.); dongjianzhang@mail.nwpu.edu.cn (D.Z.); gaomeng891023@163.com (M.G.); 2 HONGDU Aviation Industry Group Co., Ltd., Aviation Industry Corporation of China, Nanchang 330024, China; pcpc10@mail.nwpu.edu.cn

**Keywords:** polypropylene-fiber-reinforced foamed concrete, high temperature, split-Hopkinson pressure bar, high strain rate, failure mode

## Abstract

Polypropylene-fiber-reinforced foamed concrete (PPFRFC) is often used to reduce building structure weight and develop engineering material arresting systems (EMASs). This paper investigates the dynamic mechanical properties of PPFRFC with densities of 0.27 g/cm^3^, 0.38 g/cm^3^, and 0.46 g/cm^3^ at high temperatures and proposes a prediction model to characterize its behavior. To conduct the tests on the specimens over a wide range of strain rates (500~1300 s^−1^) and temperatures (25~600 °C), the conventional split-Hopkinson pressure bar (SHPB) apparatus was modified. The test results show that the temperature has a substantial effect on the strain rate sensitivity and density dependency of the PPFRFC. Additionally, the analysis of failure models demonstrates that with the melting of polypropylene fibers, the level of damage in PPFRFC under dynamic loading increases, resulting in the generation of a greater number of fragments.

## 1. Introduction

Polypropylene-fiber-reinforced foamed concrete (PPFRFC) is made by adding air bubbles and polypropylene (PP) fibers to the prepared cement mortar. This results in a lower density and excellent buffering performance due to its unique microstructural morphology. In the field of construction, the utilization of PPFRFC yields substantial benefits, such as a notable reduction in the structural dead load, resulting in a corresponding decrease in the cross-sectional dimensions of structural elements and the foundation size [1]. Taking into account the pronounced influence of structural weight on seismic acceleration and magnitude, the implementation of PPFRFC has also been demonstrated to play a notable role in mitigating the risk of earthquake damage [2]. PPFRFC is also widely used in buffer protection, particularly in the development of engineering material arresting systems (EMASs) at the end of airport runways [3]. Significant impact loads will be generated during aircraft landings, and in certain emergency situations, the aircraft may catch fire, which necessitates an emergency landing [4]. Therefore, the Federal Aviation Administration (FAA) recommends using an EMAS at the end of the runway to create a runway safety zone [5]. Considering the extensive application of PPFRFC in these conditions of high temperature and high strain rate, it is necessary to study its dynamic mechanical properties at high temperatures.

Foamed concrete exhibits nonlinear behavior comparable to that observed in metal and polymer foams, both of which possess typical three-stage deformation characteristics [6]. Due to its unique microstructure, the performance of foamed concrete is mainly affected by its porosity and pore size [7]. It has been demonstrated in the academic literature that foamed concrete with small and uniform pore diameters can attain higher strength [8]. The incorporation of fibers has been demonstrated to be a common approach to enhancing the performance of concrete-like materials, which can increase their load-carrying capacity and limit crack propagation [9]. PP fibers have demonstrated their efficacy in enhancing the residual strength of concrete [10]. Adding fibers to the production of foamed concrete is also an effective improvement method [11]. Tang et al. [12] revealed that fibers could significantly enhance the loading capacity of the pore structure. Yang et al. [13] demonstrated that the incorporation of polypropylene (PP) fibers had a significant influence on the strength of foamed concrete and altered its failure mode toward a quasi-plastic behavior. The ability of foamed concrete reinforced by fibers to resist compression and bending loads has also been extensively studied [14,15,16], but primarily under quasi-static conditions.

In recent years, growing attention has been drawn to the mechanical properties of foamed concrete subjected to dynamic loading. Guo et al. [17] studied the mechanical response of foamed concrete under low strain rates, and the results indicated that the foamed concrete was slightly sensitive to the strain rate. Deng et al. [18] demonstrated that as the strain rate increased, the strength and elastic modulus of foamed concrete significantly increased. Wang et al. [19] revealed the influence of failure modes on the impact behavior of foamed concrete. The research indicated that foamed concrete was much more sensitive to the strain rate in the shear failure mode than in the splitting failure mode. Feng et al. [20] concluded that high-strain-rate loading could significantly increase the overall level of damage to foamed concrete. Previous studies have almost entirely concentrated on strain rates less than 500 s^−1^, while there has been a limited focus on the dynamic mechanical properties of foamed concrete under a strain rate above 500 s^−1^. For PPFRFC, such research is even rarer.

Foamed concrete is a complex material containing cement slurry, aggregates (sand or small stone), and pores. Its degradation mechanism is caused by the deprivation of cement slurry, which is significantly affected by high temperatures [21]. After being exposed to high temperatures, foamed concrete undergoes a loss in mass, an increase in porosity, and the development of new cracks, each of which can detrimentally affect its overall performance [22,23]. In addition, for foamed concrete reinforced by PP fibers, the PP fibers will melt after being subjected to high temperatures, which will also adversely affect its performance. Guo et al. [24] concluded that the compressive performance of foamed concrete reinforced by PP fibers was more affected by the temperature than by the strain rate. It is worth noting that this conclusion was drawn from tests performed on a range of strain rates from 0.001 s^−1^ to 118 s^−1^ and a range of temperatures from −50 °C to 70 °C. In fact, the strength loss of foamed concrete mainly occurs above 90 °C [21], while the exploration of its mechanical response under dynamic loading has rarely been carried out above this temperature. It is inevitable that the PPFRFC will be subjected to impact loading under extreme conditions, such as high temperatures, in engineering applications. Therefore, it is essential to conduct a thorough investigation into the mechanical properties and failure modes of PPFRFC at a high temperature and high strain rate.

As stated above, previous researchers focused on the dynamic mechanical properties of PPFRFC at room temperature. The investigation of the dynamic mechanical performance of PPFRFC at high temperatures is a specific gap in the field due to the inherent challenges of conducting large-deformation dynamic tests on foam materials at high temperatures. Consequently, there is currently a lack of understanding of the compressive behavior of PPFRFC under the coupling effects of a high strain rate and high temperature. This paper aims to provide a comprehensive assessment of the compressive mechanical properties of PPFRFC under conditions of high temperature and high strain rate utilizing a modified split-Hopkinson pressure bar (SHPB) apparatus. Based on the experimental results, prediction models accounting for the coupling effects of the temperature and strain rate were developed to estimate the initial peak stress, plateau stress, and elastic modulus. These findings can serve as a valuable reference for the potential application of PPFRFC in the construction and buffer protection fields.

## 2. Materials and Experimental Methods

### 2.1. Materials and Specimens

The fundamental components of PPFRFC include cement, water, and fly ash. In this study, P·O 42.5-grade ordinary Portland cement according to the Chinese standard (GB175-2007) was used, along with a class-F type of fly ash based on the Chinese standard (GB/T1596-2017) and common tap water. PP fibers with lengths ranging from 6 to 9 mm, diameters ranging from 5 to 48 μm, and melting points ranging from 170 °C to 190 °C were incorporated. Their density, tensile strength, elasticity modulus, elongation at break, and melt flow index are 0.9 g/cm^3^, 498 MPa, 3700 MPa, 25%, and 12 g/10 min, respectively. The foaming agent comprises 12% α-olefin sulfonate (AOS) foaming agent, 1% lauryl alcohol, 13% rosin soap liquid, 2% gelatin, 5% acrylic emulsion, 2% cement sealant, and 65% water. These constituents were blended and introduced into the reactor for stirring, thus producing the desired foaming agent. Table 1 provides the composition and proportion of specimens with densities of 0.27 g/cm^3^, 0.38 g/cm^3^, and 0.46 g/cm^3^.

The production process of the standard specimen was as follows. Firstly, the foaming agent and water were mixed into a diluted liquid in a specified ratio and stirred thoroughly. The liquid mixture was then added to the foaming system to generate foam. Next, the water, cement, PP fibers, and fly ash were mixed into a uniform slurry, and then the slurry and foam were fed into the mixing system simultaneously. Finally, the foam slurry was poured into the mold, which had been coated with a release agent. The foam slurry was evenly distributed within the mold using a vibration table. Following a 3-day curing period at room temperature, the PPFRFC was removed from the mold and transferred to a cabinet that could bear a constant relative humidity of 90% for 28 days.

According to previous studies, the specimen size is recommended to be sufficiently large to accommodate an appropriate number of pores due to the strict requirements for foam materials in mechanical testing [25]. Andrews [26] proposed that in quasi-static compression and tension experiments, the foam should have at least seven pores along the thickness direction to avoid boundary effects. The specimens in this paper exhibited a range of pore diameters from 0.15 to 1.5 mm. Therefore, the specimens used in this study were designed as cylinders with dimensions of Φ45 × 15 mm to improve the loading strain rate.

### 2.2. Experimental Apparatus and Methods

#### 2.2.1. Modified SHPB Apparatus at High Temperatures

There are two main methods for testing the high-temperature impact response of concrete-like materials. One is to directly load the specimen once it reaches an elevated temperature, while the other one is to carry out the compression test when the specimen is cooled to room temperature [27]. Considering that uneven shrinkage during the cooling process can affect the specimen, the tests in this paper were conducted using the “at elevated temperature” method.

**Figure 1:**
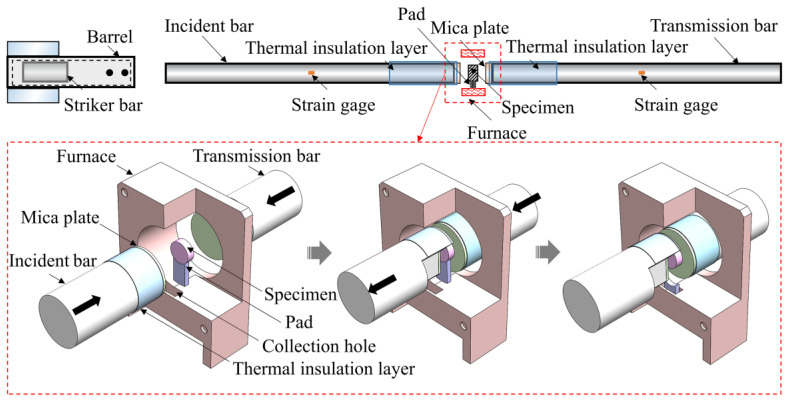
Diagram of the high-temperature furnace and large-diameter nylon SHPB system.

To conduct the large-deformation dynamic testing of specimens at high temperatures, some improvements were proposed, as shown in Figure 1. Firstly, a large elastic deformation in an incident bar is necessary to compress the foam specimen to the densification stage, which requires a long-duration incident wave [28]. However, traditional loading bars made of metal materials have a high elastic modulus and wave velocity, which limits the wave duration. Additionally, the metal loading bar has a higher acoustic impedance compared to the foam specimen, making it challenging to achieve a satisfactory signal-to-noise ratio [29]. Therefore, a nylon bar with a diameter of 100 mm was employed as a loading bar to generate a long-duration incident wave. Nylon, as a viscoelastic material, has a lower elastic modulus and wave velocity compared to metal, and its acoustic impedance is comparable to that of PPFRFC. Its density, elastic modulus, and acoustic impedance are 1.14 g/cm^3^, 2.5 GP, and 1.71 MPa·m^−1^·s^−1^, respectively. Secondly, a pair of mica plates were mounted on the bar–specimen interface to prevent it from being heated. A thermal insulation layer was axially wrapped around the portion of the nylon bar adjacent to the specimen. To position the specimen in the middle of the bar–specimen interface and evenly distribute the force, a pad was added to support the specimen. During the heating process, the loading bars were kept separate from the heating area. After heating the specimen to the specified temperature and maintaining it for a certain period, the two loading bars were pushed toward the specimen until they contacted it. Then, the loading bars and the specimen were pushed to the left until the pad fell into the collection hole. The above process was controlled within 5 s. Finally, the striker bar was launched to impact the incident bar, which generated an incident wave to load the specimen.

#### 2.2.2. Heating of Specimens

**Figure 2:**
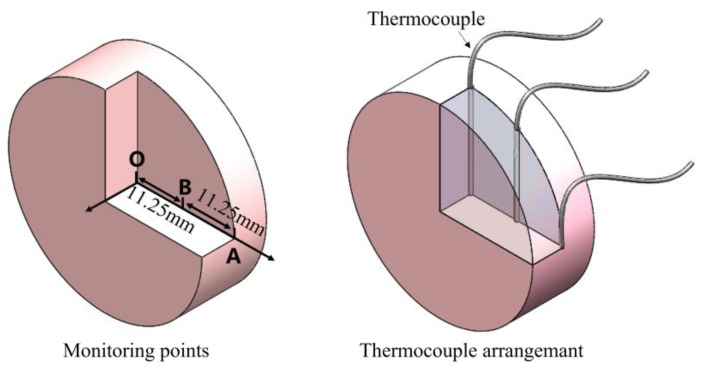
Schematic diagram of temperature monitoring points and thermocouple arrangement for the specimen.

The specimens were heated to a specified high temperature in the tests using a radiant-heating furnace. The furnace was able to be maintained at a constant temperature within ±5 °C. Owing to the large dimensions and limited ability to conduct the heat of the foam material, the surface area and the center area of the specimen require different times to be heated to the specified temperature. Therefore, the time required for specimens with densities of 0.27 g/cm^3^, 0.38 g/cm^3^, and 0.46 g/cm^3^ to reach a uniform temperature condition should be determined before the test. Three temperature measuring points (points A, B, and O) were selected along the radial direction of the specimen, as shown in Figure 2. Point O is in the center of the specimen, while the lengths of OA and OB are 22.5 mm and 11.25 mm, respectively. Thermocouples were employed to monitor the temperature at these points. At point A, the thermocouple was fixed by binding, while at points B and O, it was embedded in holes drilled beforehand in the specimen, and these holes were finally filled with cement.

#### 2.2.3. Correction of Wave Dispersion

Given the large diameter of Φ100 mm nylon bars, geometric dispersion is unavoidable; therefore, the influence of wave dispersion must be considered. Additionally, the viscoelastic characteristics of nylon bars will also cause dispersion and damping. Therefore, the propagation coefficient method [30,31] was adopted to correct the wave dispersion.

### 2.3. Validity Analysis of the Modified High-Temperature Dynamic Test Method

#### 2.3.1. Influence of the Thickness of Mica Plate on Wave Propagation

A mica plate is a material that has excellent performance in resisting high temperatures and preventing heat conduction when used as a heat insulation plate. Its acoustic impedance is close to that of nylon, which can effectively reduce the interference of wave propagation. The density, elastic modulus, and acoustic impedance of the mica plate were 2.2 g/cm^3^, 1.5 GPa, and 1.82 MPa·m^−1^·s^−1^, respectively. To evaluate the disturbance of mica plates on the propagation of the wave, some impact tests were performed. These tests comprised two sets of conditions: one without the mica plate and the other utilizing mica plates with a diameter of 100 mm and various thicknesses of 5 mm, 10 mm, 15 mm, and 20 mm. The incident bar was impacted by the striker bar at 7 m/s.

**Figure 3:**
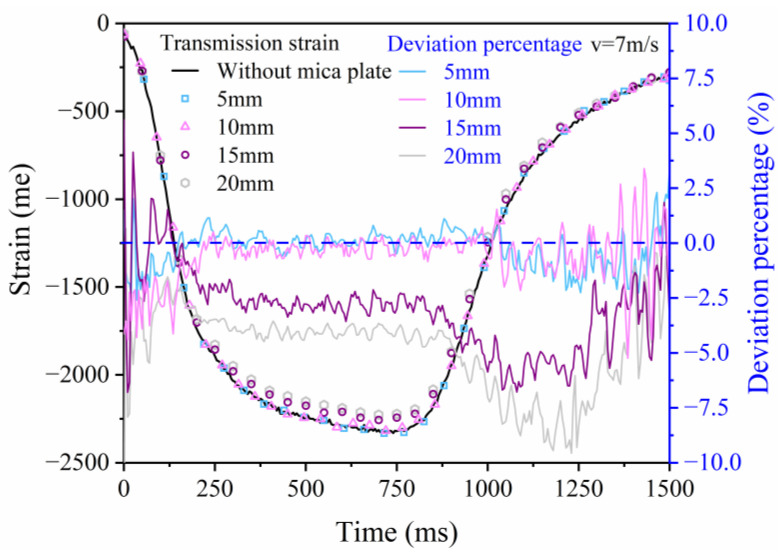
Influence of mica plates with thicknesses of 5 mm, 10 mm, 15 mm, and 20 mm on wave propagation.

The transmission strains were collected, and the deviation percentages in the transmission strain were calculated as follows [32]:

(1) $$\Delta \varepsilon =\left(\varepsilon_{m}-\varepsilon_{n}\right)/\varepsilon_{n}\times 100\%,$$

where $\varepsilon_{n}$ is the transmission strain without the mounting of the mica plate, and $\varepsilon_{m}$ is the transmission strain after mounting the mica plate. Figure 3 shows that the deviation percentages of mica plates with thicknesses of 20 mm and 15 mm are 9.5% and 6.7%, respectively. In contrast, for the 10 mm and 5 mm mica plates, their maximum deviation percentages are 5.1% and 2.4%, respectively. These indicate that the error decreases as the thickness decreases, and the influence of a mica plate with a thickness of 5 mm on wave propagation can be ignored. However, to ensure that the nylon bar is within the operating temperature range during the test, the thermal insulation effect of the mica plates with thicknesses of 5 mm, 10 mm, 15 mm, and 20 mm also needs to be evaluated, which will be discussed in the next section.

#### 2.3.2. Thermal Insulation Performance of Mica Plate

**Figure 4:**
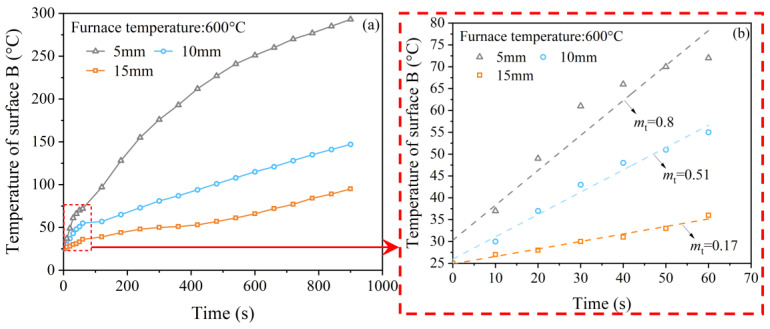
Time–temperature curves for mica plates with thicknesses of 5 mm, 10 mm, and 15 mm. (**a**) Time–temperature curves and (**b**) linear fitted analysis of the time-temperature curves within the initial 60 s.

To evaluate the insulation effect of mica, some tests were carried out. First, the surface of the mica plate subjected to heat was defined as surface A. The other surface that experienced room temperature was defined as surface B and was covered with thermally insulated cotton. Then, each mica plate was positioned horizontally on the upper surface of the heated furnace, and the furnace temperature was kept at 600 °C. A thermocouple was positioned at the center of surface B to monitor its temperature development. Figure 4 shows the time–temperature curves of surface B for each thickness. The curves in Figure 4b were linearly fitted, and the slope was calculated to represent the heating rate in the first 60 s. The slopes for specimens with thicknesses of 5 mm, 10 mm, and 15 mm were 0.8, 0.51, and 0.17, respectively. The temperatures of the mica plate at the 5th second were 34 °C (5 mm), 28 °C (10 mm), and 25 °C (15 mm), respectively. In this study, the assembly time of the loading bar and specimen was controlled within 5 s. As a result, the mica plate with a thickness of 5 mm could meet thermal insulation requirements without interfering with wave propagation.

### 2.4. Experimental Scheme

Firstly, the PPFRFC was sectioned into standard specimens measuring Φ45 × 15 mm. Subsequently, the specimens were categorized and assigned individual numbers based on their respective densities. Finally, a series of dynamic compression tests were conducted on the PPFRFC specimens with three different densities (0.27 g/cm^3^, 0.38 g/cm^3^, and 0.46 g/cm^3^) at various temperatures (25 °C, 200 °C, 400 °C, and 600 °C) and strain rates (500 s^−1^, 700 s^−1^, 900 s^−1^, and 1300 s^−1^). To ensure the consistency of the results, each condition was tested three to five times.

### 2.5. Characteristic Indicators

To quantitatively analyze the properties of the material, several parameters were defined. The initial peak stress $\sigma_{i}$ is a crucial parameter in evaluating the loading capacity of materials. It is the maximum stress value of the specimen obtained prior to the plateau stage. The plateau stress, denoted as $\sigma_{p}$, corresponds to the average stress level experienced during the plateau stage. The densification stage commences at a critical strain value, which is characterized as the densification strain $\varepsilon_{d}$. An approach based on energy [17] was employed to determine the values of $\sigma_{p}$ and $\varepsilon_{d}$. The energy absorption efficiency η(ε) represents the ratio of the total energy absorbed before reaching the given nominal strain ε to the corresponding stress σ(ε), which can be calculated as follows [17]:

(2) $$\eta \left(\varepsilon \right)=\int_{0}^{\varepsilon }{\sigma \left(\varepsilon \right)}_{d}/\sigma \left(\varepsilon \right),$$

The densification strain $\varepsilon_{d}$ corresponds to the strain when the energy absorption efficiency reaches its maximum value, which can be defined as [17]

(3) $${\left.\left[d\eta \left(\varepsilon \right)/d\varepsilon \right]\right|}_{\varepsilon =\varepsilon_{d}}=0.$$

The plateau stress can be expressed as [17]

(4) $$\sigma_{p}=\left[1/\left(\varepsilon_{d}-\varepsilon_{i}\right)\right]\int_{\varepsilon_{i}}^{\varepsilon_{d}}\sigma \left(\varepsilon \right)d\varepsilon ,$$

where $\varepsilon_{i}$ is the strain at the initial peak stress.

Subsequently, the parameters $\sigma_{i}$, $\sigma_{p}$, and $\varepsilon_{d}$ at different temperatures (25 °C, 200 °C, 400 °C, and 600 °C) and strain rates (500 s^−1^, 700 s^−1^, 900 s^−1^, and 1300 s^−1^) were calculated according to the above method. The specific parameters are listed in Table 2, Table 3 and Table 4.

## 3. Results and Discussion

### 3.1. Microscopic Morphology of PPFRFC Specimens

**Figure 5:**
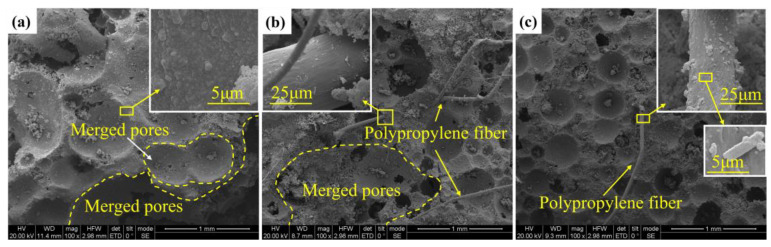
Scanning electron micrograph of PPFRFC at 25 °C with densities of (**a**) 0.27 g/cm^3^; (**b**) 0.38 g/cm^3^; and (**c**) 0.46 g/cm^3^.

The SEM micrographs of the PPFRFC at 25 °C are illustrated in Figure 5. As shown in Figure 5a, there are numerous irregular pores inside the specimen with a density of 0.27 g/cm^3^. Some pores merge into large-sized pores (Figure 5a,b). As the density increases, the pore structure becomes orderly and regular and has a uniform spherical distribution, as shown in Figure 5c. This can be attributed to the varying water-to-cement ratios employed in specimens with different densities. The water-to-cement ratio plays a crucial role in the production of PPFRFC, as it affects not only the density but also the pore structure, thereby influencing the mechanical properties of the material [33]. In comparison to high-density specimens, low-density specimens possess a higher water-to-cement ratio. Increasing the water-to-cement ratio results in a decrease in the density and compressive strength of the PPFRFC [34]. As the water-to-cement ratio increases, the size of the pores is progressively enlarged, and the surface tensile force of the cement paste decreases. Consequently, the pores suffer severe damage, leading to a significant reduction in the uniformity of the pore size [35].

**Figure 6:**
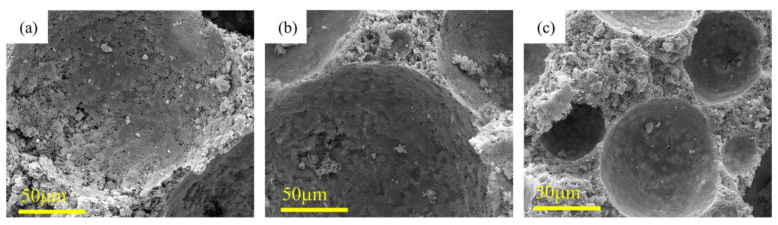
Scanning electron micrograph of PPFRFC at 600 °C with densities of (**a**) 0.27 g/cm^3^; (**b**) 0.38 g/cm^3^; and (**c**) 0.46 g/cm^3^.

Figure 6 depicts the SEM micrographs of the PPFRFC at 600 °C with densities of 0.27 g/cm^3^, 0.38 g/cm^3^, and 0.46 g/cm^3^. At 600 °C, the pore walls of the specimen with a density of 0.27 g/cm^3^ exhibit a significant number of cracks and pores. This leads to an increase in pore connectivity within the specimen, thereby exacerbating the stress concentration under loading conditions. In contrast, the pore wall structure of the specimen with a density of 0.46 g/cm^3^, although becoming looser, still maintains a relatively dense surface with only a few cracks, indicating that these pores retain good air tightness.

### 3.2. Stress–Strain Response Curves

**Figure 7:**
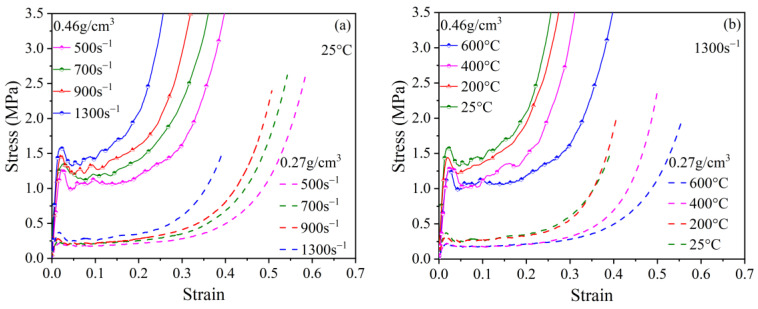
Dynamic compressive stress–strain curves of PPFRFC with different densities under various loading conditions: (**a**) at a temperature of 25 °C and various strain rates; (**b**) at a strain rate of 1300 s^−1^ and various temperatures.

**Figure A1:**
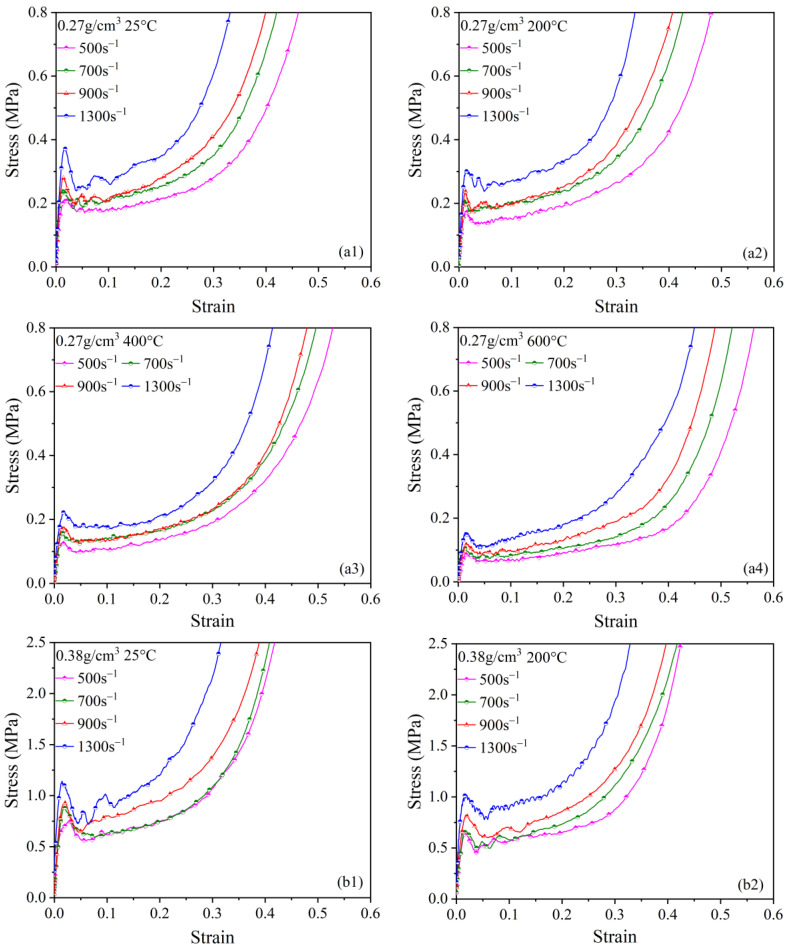

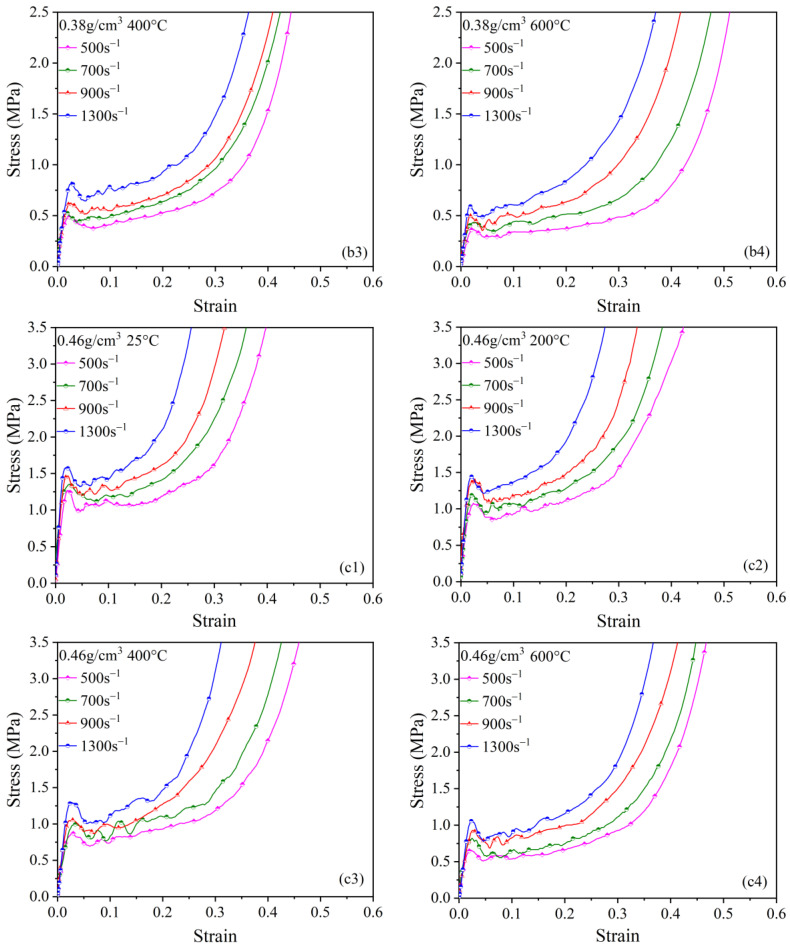
Typical three-stage deformation characteristics in dynamic compressive stress–strain curves of PPFRFC with different densities at various temperatures: (**a1**–**a4**) 0.27 g/cm^3^; (**b1**–**b4**) 0.38 g/cm^3^; (**c1**–**c4**) 0.46 g/cm^3^.

Figure 7 presents the representative compressive stress–strain curves of specimens with densities of 0.27 g/cm^3^ and 0.46 g/cm^3^ at both 25 °C and 600 °C, with strain rates ranging from 500 s^−1^ to 1300 s^−1^. For more detailed information, refer to Figure A1 in Appendix A. Here, representative stress–strain curves among the curves from the repeatability test were selected. These curves show distinctive three-stage deformation characteristics: the linear elastic stage, the plateau stage, and the densification stage. In the initial loading stage, the stress–strain relationship of the PPFRFC is linear. When the PPFRFC is further compressed, plastic deformation occurs. Then, the walls of the pores begin to bear the load, creating a stable stress level with occasional fluctuations. After all the pores have been compacted, the PPFRFC enters the densification stage, which is characterized by a marked increase in stress as the strain level increases. Figure 7 clearly illustrates that as the strain rate increases, there is an improvement in the strength of the PPFRFC. However, the strength of the PPFRFC is weakened as the temperature rises when tested at the selected strain rate. The figure also illustrates that a rise in density leads to an increase in strength at a specified temperature and strain rate.

### 3.3. Dynamic Mechanical Properties of PPFRFC at High Temperatures

**Figure 8:**
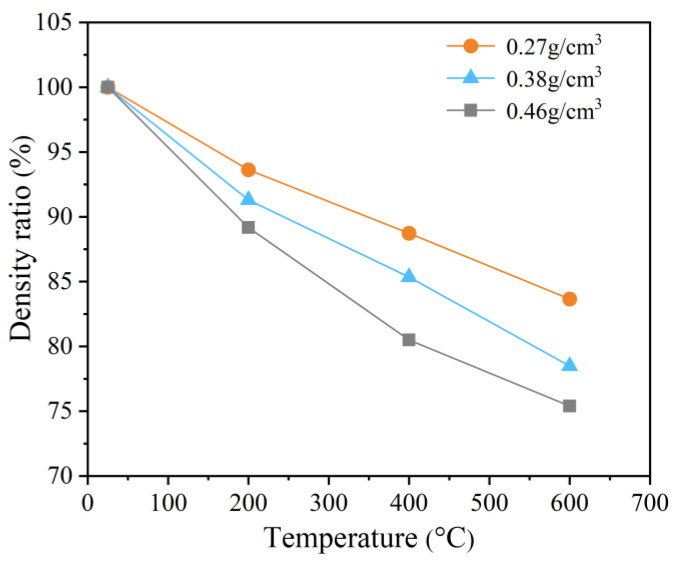
Original density percentage of specimens at temperatures of 25 °C, 200 °C, 400 °C, and 600 °C.

Under conditions of high temperature, the evaporation of both the free and bound water within the specimen will result in significant changes in its mass and density. To quantify the extent of the density reduction, the density ratio was calculated using the following equation [22]:

(5) $$\alpha =\rho_{2}/\rho_{1}\times 100\%,$$

where α is the density ratio, and $\rho_{1}$ and $\rho_{2}$ are the densities of the PPFRFC before and after experiencing different high temperatures. The results are depicted in Figure 8.

Figure 8 shows that the decline in the density of the specimen with a higher density, which contains a significantly higher proportion of both free and chemically bonded water, is more pronounced in comparison to the specimen with a lower density. The density ratios of the specimens with densities of 0.27 g/cm^3^, 0.38 g/cm^3^, and 0.46 g/cm^3^ decrease by 16%, 22%, and 25%, respectively, as the temperature gradually rises from 25 °C to 600 °C.

#### 3.3.1. Influence of Density on Initial Peak Stress, Plateau Stress, and Elastic Modulus

**Figure 9:**
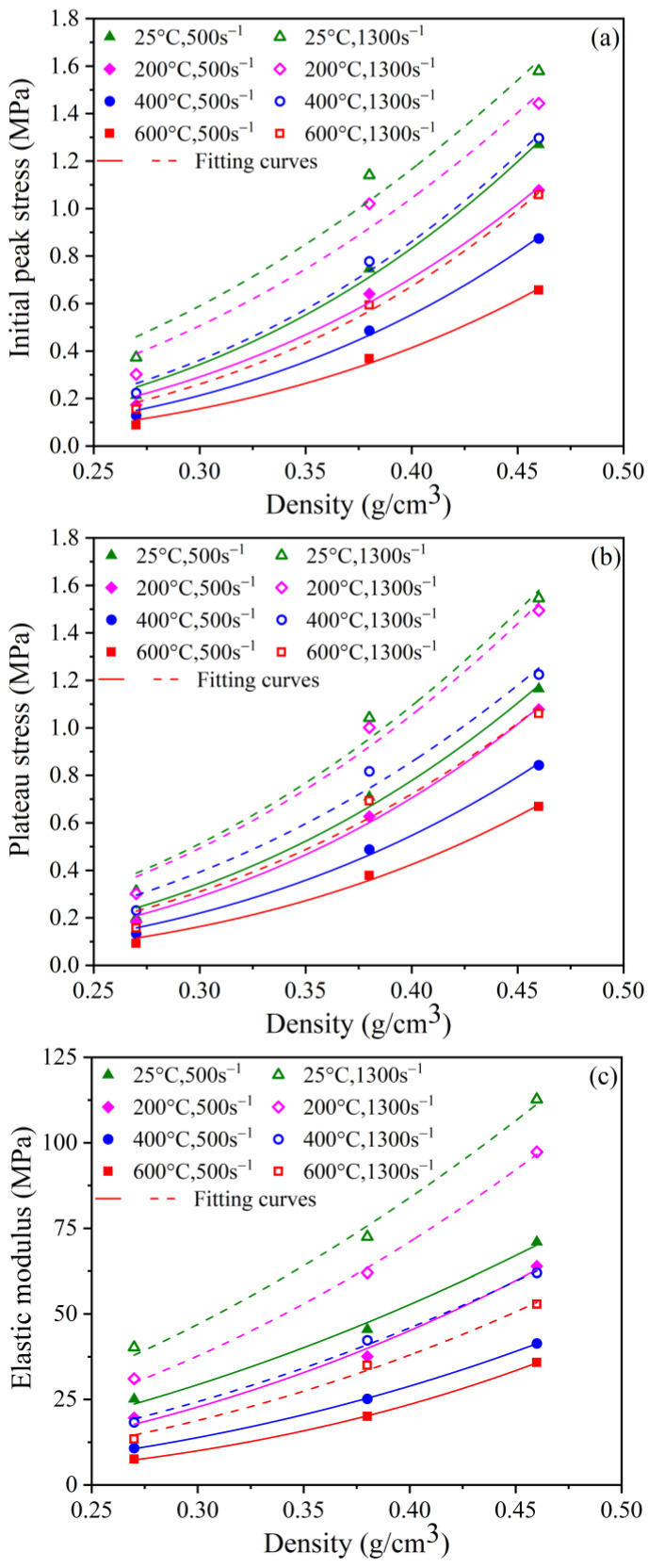
Empirical fitting of initial peak stress, plateau stress, and elastic modulus versus density at different strain rates and temperatures. (**a**) Initial peak stress; (**b**) plateau stress; and (**c**) elastic modulus.

Figure 9 depicts the initial peak stress $\sigma_{i}$, plateau stress $\sigma_{p}$, and elastic modulus E as a function of density at various strain rates and temperatures. These values are improved with increasing density for a given strain rate. However, this trend will become flat as the temperature rises. This implies that the sensitivity of the PPFRFC to the density is influenced by the temperature. The fundamental explanation is that specimens with a greater density have smaller pore diameters and sturdier pore walls, enhancing the pore walls’ resistance to bending and compression, as observed in prior studies [20]. When the temperature rises, the pore walls become brittle and cracked due to dehydration, resulting in a decreased ability to withstand external loads. The high-density specimens are more noticeably affected, which causes a more significant reduction in strength compared to the low-density specimens. When the temperature is the same, the dependence of the PPFRFC specimens on the density improves as the strain rate increases.

The following functions were introduced to quantify the correlation between the PPFRFC specimens and density.

(6) $$\left\{\begin{array}{c} \sigma_{i}=A_{i}{\left(\rho /\rho_{0}\right)}^{m_{i}}\\ \sigma_{p}=A_{p}{\left(\rho /\rho_{0}\right)}^{m_{p}}\\ E=A_{e}{\left(\rho /\rho_{0}\right)}^{m_{e}} \end{array},\right.$$

where $\rho_{0}$ is the reference density, taken as 0.27 g/cm^3^, and ρ is the density. *A*_i_, *A*_p_, *A*_e_, *m*_i_, *m*_p_, and *m*_e_ are the fitting parameters obtained from experimental data, as indicated in Table 5. The fitting curves presented in Figure 9 indicate that $\sigma_{i}$, $\sigma_{p}$, and E follow a power-law relationship with ρ.

#### 3.3.2. Influence of Density on Densification Strain

**Figure 10:**
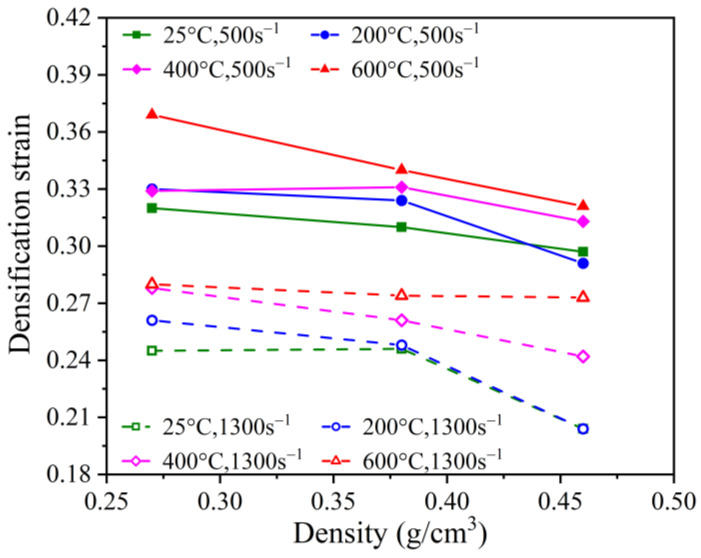
Relationship between densification strain and density at strain rates of 500 s^−1^ and 1300 s^−1^.

A graphical representation of the correlation between the density and the densification strain is presented in Figure 10. At a strain rate of 500 s^−1^, the densification strains for temperatures of 25 °C, 200 °C, 400 °C, and 600 °C decrease by 7.2%, 11.8%, 4.9%, and 13%, respectively, as the density increases from 0.27 g/cm^3^ to 0.46 g/cm^3^. At a strain rate of 1300 s^−1^, the densification strains decrease by 16.7%, 21.8%, 12.9%, and 2.5%, respectively. This shows a negative density dependence for the densification strain. However, at high strain rates, the dependence of the densification strain on the density decreases significantly with the increase in temperature. This can be attributed to the densification mechanism of PPFRFC. The densification of PPFRFC is caused by the compaction of pores. Prior to pore compaction, the load in a specimen is primarily carried by the pore walls, resulting in a smaller load-bearing area and a lower bearing capacity. After the pore walls are crushed, they fill the pores, increasing the load-bearing area of the specimen and consequently enhancing its carrying capacity. Under high-strain-rate loading, the pore walls collide before being fully crushed. At this stage, due to the dense structure of the pore walls, they still possess significant strength, resulting in the specimen entering the densification stage early. In comparison to low-density specimens, high-density specimens have smaller pores and stronger pore walls, making this phenomenon more pronounced. After being subjected to high temperatures, the pore wall structure of the specimen becomes looser and more brittle. Even when tightly packed, the pore wall cannot provide a sufficient bearing capacity. As a result, under high-strain-rate conditions, the dependence of the densification strain on the density diminishes as the temperature rises.

#### 3.3.3. Influence of the Strain Rate on Initial Peak Stress, Plateau Stress, and Elastic Modulus

To reveal the temperature effect on PPFRFC specimens, the initial peak stress ratio $\gamma_{i}$, plateau stress ratio $\gamma_{p}$, and elastic modulus ratio $\gamma_{e}$ were employed, which are defined by the following equation:

(7) $$\left\{\begin{array}{c} \gamma_{i}=\sigma_{i2}/\sigma_{i1}\\ \gamma_{p}=\sigma_{p2}/\sigma_{p1}\\ \gamma_{e}=E_{2}/E_{1} \end{array},\right.$$

where $\sigma_{i1}$, $\sigma_{p1}$, and $E_{1}$ are the values of the initial peak stress, plateau stress, and elastic modulus of PPFRFC at 25 °C, while $\sigma_{i2}$, $\sigma_{p2}$, and $E_{2}$ are those at different high temperatures, respectively.

**Figure 11:**
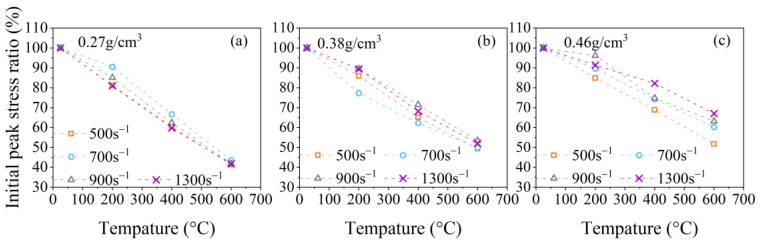
Temperature effects on the initial peak stress ratio for specimens with three densities at different strain rates: (**a**) 0.27 g/cm^3^; (**b**) 0.38 g/cm^3^; (**c**) 0.46 g/cm^3^.

**Figure 12:**
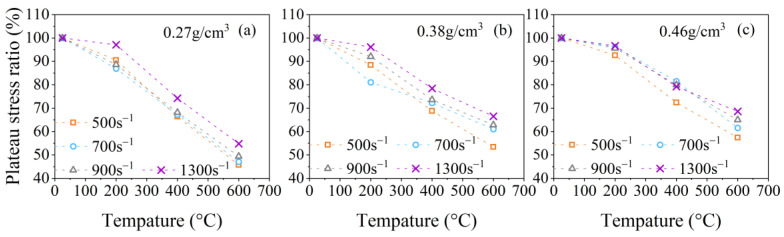
Temperature effects on the plateau stress ratio for specimens with three densities at different strain rates: (**a**) 0.27 g/cm^3^; (**b**) 0.38 g/cm^3^; (**c**) 0.46 g/cm^3^.

**Figure 13:**
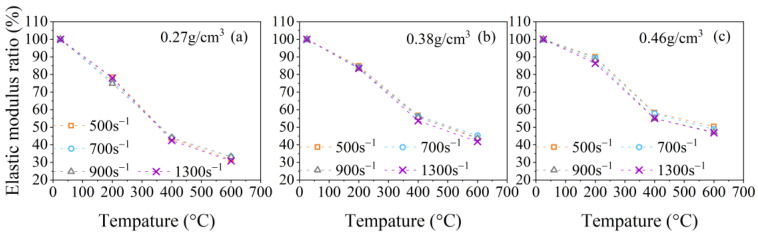
Temperature effects on the elastic modulus ratio for specimens with three densities at different strain rates: (**a**) 0.27 g/cm^3^; (**b**) 0.38 g/cm^3^; (**c**) 0.46 g/cm^3^.

**Figure 14:**
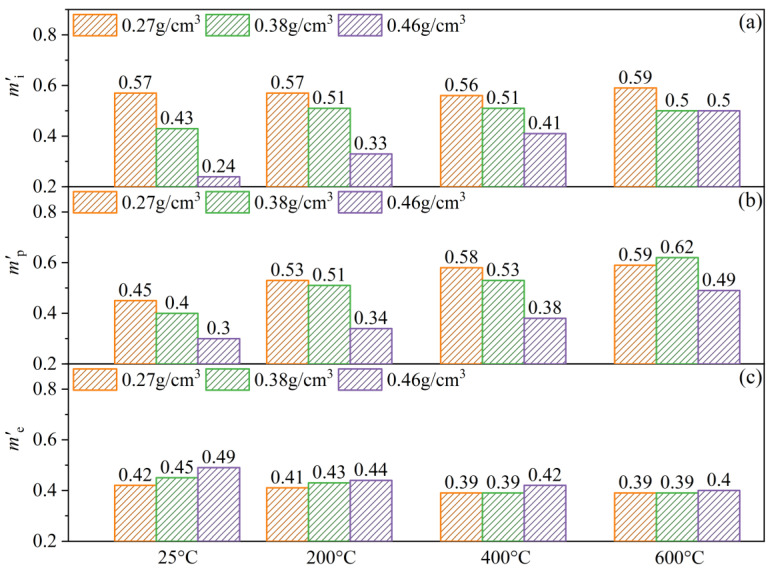
Strain rate sensitivity coefficients of PPFRFC with three densities at temperatures of 25 °C, 200 °C, 400 °C, and 600 °C. (**a**) Initial peak stress; (**b**) plateau stress; and (**c**) elastic modulus.

The relationships between $\gamma_{i}$, $\gamma_{p}$, and $\gamma_{e}$ and the temperature at strain rates of 500 s^−1^, 700 s^−1^, 900 s^−1^, and 1300 s^−1^ are presented in Figure 11, Figure 12, Figure 13 and Figure 14. It is evident from Figure 11a–c that the initial peak stress ratio $\gamma_{i}$ of PPFRFC with all densities decreases monotonously as the temperature rises. Figure 12a–c show that the plateau stress ratio $\gamma_{p}$ of specimens with all densities experiences a steeper decrease from 200 °C to 400 °C compared to the decrease from 25 °C to 200 °C. One of the reasons for the decrease in the plateau stress ratio $\gamma_{p}$ at a temperature range of 25 °C to 200 °C can be attributed to the diminished cohesion of the van der Waals forces among the calcium silicate hydrate layers [22]. The surface energy of hydrated calcium silicate is thus reduced, and silanol groups (Si-OH: OH-Si) are formed, which exhibit a weaker bond strength [36]. An additional contributing factor is the formation of new pores during the melting of PP fibers. The newly formed pores exacerbate the structural damage, which decreases the overall strength of the PPFRFC [27]. During the temperature increase from 200 °C to 400 °C, the decomposition of the C-S-H gel and the sulfoaluminate phase causes the pore walls to become brittle and more easily crack. These cracks severely influence the structural integrity of the PPFRFC, which causes a more pronounced decrease in the plateau stress ratio $\gamma_{p}$ [37]. As the temperature rises from 400 °C to 600 °C, the decreasing trends of the plateau stress ratio $\gamma_{p}$ diminish. The relationship of the plateau stress ratio $\gamma_{p}$ with temperature also applies to the elastic modulus ratio $\gamma_{e}$, as shown in Figure 13a–c.

To quantify the effect of the strain rate, linear fits of the logarithmic relationships between the initial peak stress, plateau stress, and elastic modulus and the strain rate were performed for PPFRFC at temperatures of 25 °C, 200 °C, 400 °C, and 600 °C. The values $m_{i}^{\prime }$, $m_{p}^{\prime }$, and $m_{e}^{\prime }$ are the slopes of the fitting curves, which represent the degree of sensitivity of the initial peak stress, plateau stress, and elastic modulus to the strain rate, as seen in Figure 14.

Figure 14a shows that, as the temperature rises from 25 °C to 600 °C, the value of $m_{i}^{\prime }$ increases by only 3.5% for a density of 0.27 g/cm^3^ and even experiences a slight decrease at 400 °C. When compared to 25 °C, the value of $m_{i}^{\prime }$ for a density of 0.38 g/cm^3^ increases by 16% at 200 °C, while changes little above 200 °C. However, the value of $m_{i}^{\prime }$ for a density of 0.46 g/cm^3^ is considerably affected by the temperature, increasing by 108% as the temperature rises from 25 °C to 600 °C. Additionally, the value of $m_{i}^{\prime }$ decreases as the density increases at a given temperature, which indicates a negative density dependence.

Figure 14b demonstrates that the values of $m_{p}^{\prime }$ increase with increasing temperature for all densities. For densities of 0.27 g/cm^3^, 0.38 g/cm^3^, and 0.46 g/cm^3^, the value of $m_{p}^{\prime }$ increases by 31%, 55%, and 63%, respectively, as the temperature increases from 25 °C to 600 °C. It is suggested that the strain rate sensitivity of the plateau stress has a positive temperature dependence, which is more pronounced for a density of 0.46 g/cm^3^. Additionally, it is shown that the values of $m_{p}^{\prime }$ decrease with increasing density at 25 °C, 200 °C, and 400 °C, while they first increase and then decrease at 600 °C.

The above analysis indicates that the PPFRFC specimens are sensitive to the strain rate. According to a widely recognized view, cracks within concrete-like materials have a path-altered effect when subjected to dynamic loadings, which enhances the strength of the material [19]. Due to the faster propagation speed of stress waves compared to cracks, cracks will occur randomly throughout the specimen rather than only propagating along the weakest links. As a result, the crack propagation path is altered under high strain rates, leading to the formation of more short-length cracks. These cracks consume energy from the impact load, thereby enhancing the strength of PPFRFC. The influence of the crack propagation path’s alteration on the strength of PPFRFC is more pronounced at lower densities. This means that as the temperature rises, the strain rate sensitivity of PPFRFC will be enhanced because of its decreased density at high temperatures.

Remarkably, it is imperative to recognize that the influence of gas on the compression performance of foam materials under dynamic loading should not be disregarded [38]. When subjected to loading at high strain rates, the gas trapped in the pores cannot escape in time, which helps the pore wall resist an external force. The interaction of the gas with the pore structure increases the local equivalent plastic strain and structural stress, which significantly enhances the strength of the material. As the temperature increases, the pore walls become more brittle, particularly for low-density specimens with thinner pore walls. Consequently, such specimens are more likely to crack and release gas under loading. In contrast, the higher-density specimen has smaller pores and thicker pore walls than the lower-density specimen, enabling it to maintain its airtightness at higher temperatures more effectively. Therefore, it follows that the influence of gas on the strength of specimens with a higher density is significant even when exposed to high temperatures.

In Figure 14c, the values of $m_{e}^{\prime }$ for specimens with densities of 0.27 g/cm^3^, 0.38 g/cm^3^, and 0.46 g/cm^3^ decrease by 7%, 13%, and 18%, respectively, as the temperature rises from 25 °C to 600 °C. The sensitivity of the elastic modulus to the strain rate is attributed to the delayed response of the strain to the high-velocity propagation of stress waves. For the given stress, the delayed response of the strain leads to a reduction in the strain level, thereby strengthening the elastic modulus [20]. At high temperatures, the degradation of the internal components of the specimen and the melting of PP fibers lead to the formation of new pores. These new pores significantly hinder the velocity of stress parallel to the loading direction, which weakens the delayed response of the strain. Therefore, the strain rate sensitivity of the elastic modulus is negatively dependent on temperature.

#### 3.3.4. Influence of the Strain Rate on Densification Strain

**Figure 15:**
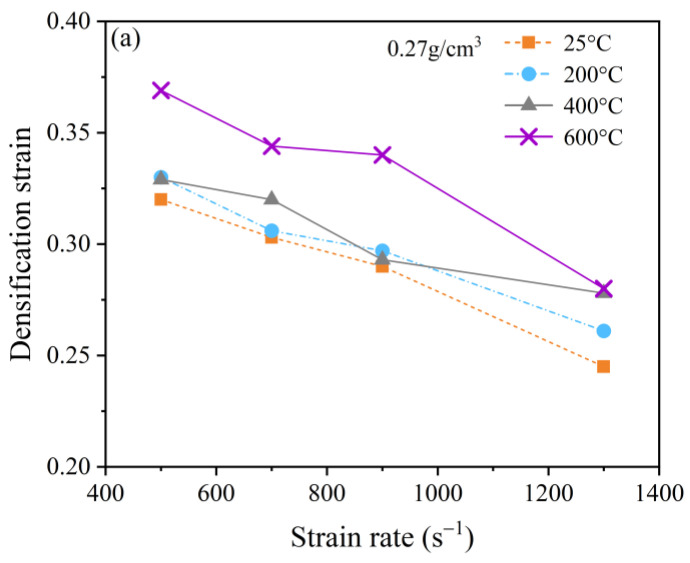

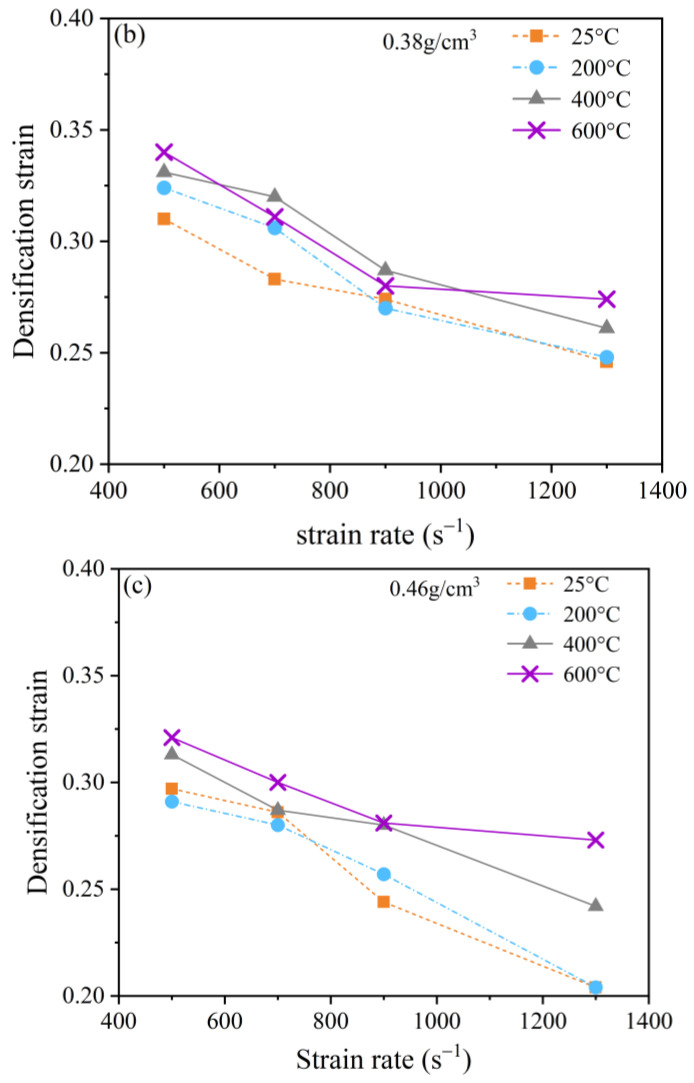
Relationship between the densification strain and the strain rate at temperatures of 25 °C, 200 °C, 400 °C, and 600 °C. (**a**) 0.27 g/cm^3^; (**b**) 0.38 g/cm^3^; and (**c**) 0.46 g/cm^3^.

Figure 15 reveals that the densification strain for the specimens decreases as the strain rate increases at various temperatures, which indicates a negative strain rate sensitivity. This is because the main deformation modes of foam materials during collapse were the buckling and bending of the pore walls. When subjected to loading at lower strain rates, the walls of the pores have sufficient time to bend and rotate, leading to the minimization of the pore space. However, as the strain rate increases, the deformed walls do not have enough time to rotate and bend before being subjected to further deformation. The result is that the walls of the pores collide before they can be completely compressed and reach their minimum size, which leads to the premature hardening of the specimen [39].

It can also be observed that the densification strain generally tends to increase as the temperature rises when the selected strain rate is the same. However, there are anomalies in that the relationship between the densification strain and temperature appears to deviate from this trend at some strain rates. For example, for the specimen with a 0.38 g/cm^3^ density, the densification strain at 400 °C is greater than that at 600 °C when the strain rates are 700 s^−1^ and 900 s^−1^. This phenomenon can be attributed to the competition between the softening and hardening of the specimen caused by the temperature and strain rate, respectively.

### 3.4. The Failure Mode of PPFRFC

**Figure 16:**
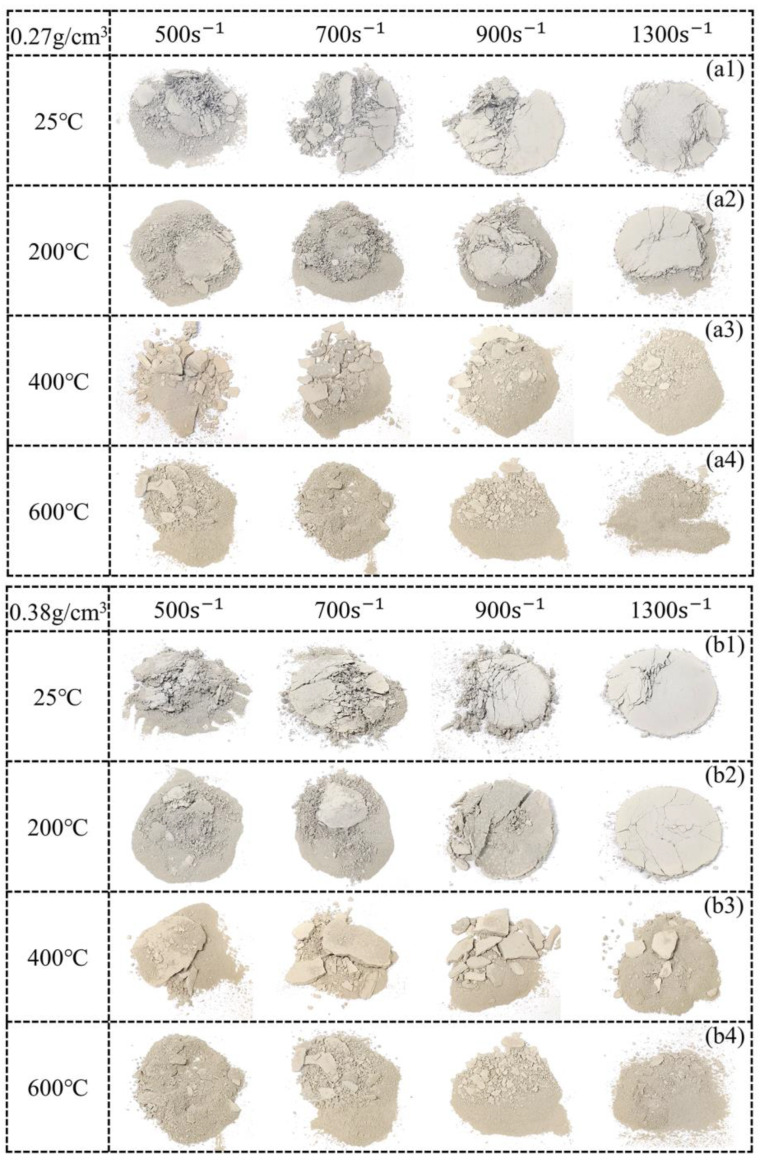

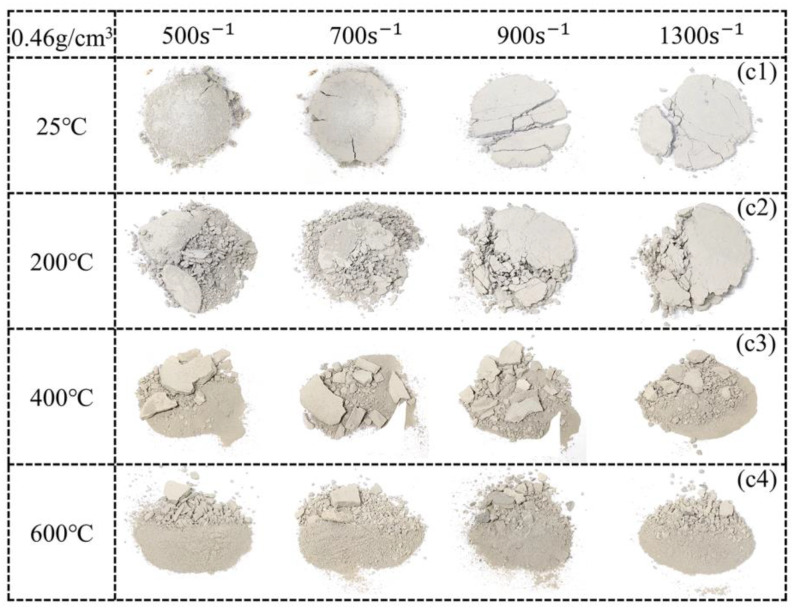
Failure modes at different strain rates and temperatures for the PPFRFC specimens with three densities: (**a1**–**a4**) 0.27 g/cm^3^, (**b1**–**b4**) 0.38 g/cm^3^, and (**c1**–**c4**) 0.46 g/cm^3^.

Figure 16 illustrates the failure modes of PPFRFC with densities of 0.27 g/cm^3^, 0.38 g/cm^3^, and 0.46 g/cm^3^ at various strain rates and temperatures. When the temperature is below 200 °C, the specimen with a density of 0.27 g/cm^3^ splits into large fragments with visible pulled fibers at a lower strain rate (500 s^−1^), as shown in Figure 16(a1,a2). As the strain rate gradually increases to 1300 s^−1^, the basic contour of the PPFRFC is relatively complete with a few cracks. It is worth mentioning that these phenomena differ from some of the results reported in the literature [23]. This is because, at high strain rates, the cracks in the specimen become much shorter, limiting the expansion of the failure region and leading to the specimen being compacted layer by layer. Simultaneously, PP fibers act as a bridge, connecting the fragments together and restraining the cracking and subsequent deformation of the specimen [10]. This results in the fragments adhering to the fibers and becoming compacted, thus maintaining a relatively complete contour of the specimen. When the temperature exceeds 200 °C, the PP fibers melt, causing the fragments to be unable to stick together. As a result, the specimen loses its basic contour when subjected to dynamic loadings, as shown in Figure 16(a3,a4). In particular, compared to specimens at temperatures below 200 °C, the fragments generated by the PPFRFC specimen consist of finer particles and more powder-like material as the strain rate increases. The failure mode for the specimen with a density of 0.27 g/cm^3^ is also observed in the specimen with a density of 0.38 g/cm^3^, as shown in Figure 16(b1–b4).

Compared to other densities at 25 °C, the PPFRFC specimen that possesses a density of 0.46 g/cm^3^ shows a different failure mode, as seen in Figure 16(c1). It can be observed in the figure that the specimen at a strain rate of 500 s^−1^ is only flattened, with only a few fragments. As the strain rate increases, cracks on the surface of the specimen begin to increase. At a strain rate of 1300 s^−1^, the PPFRFC specimen is seriously damaged, and the fragments grow in size. When the temperature exceeds 200 °C, the failure mode of the specimen closely resembles that of the specimens with densities of 0.27 g/cm^3^ and 0.38 g/cm^3^, but the fragments are significantly larger, as seen in Figure 16(c3,c4).

### 3.5. Development of Models to Predict Mechanical Properties of PPFRFC

To further study the mechanical response of PPFRFC to the temperature and strain rate, a modified prediction model is proposed in Equation (8).

(8) $$\left\{\begin{array}{c} \sigma_{i}=\sigma_{i}^{*}D_{i}\left(T\right)M_{i}\left(\dot{\varepsilon }\right)\\ \sigma_{p}=\sigma_{p}^{*}D_{p}\left(T\right)M_{p}\left(\dot{\varepsilon }\right)\\ E=E^{*}D_{e}\left(T\right)M_{e}\left(\dot{\varepsilon }\right) \end{array},\right.$$

where $\sigma_{i}^{*}$, $\sigma_{p}^{*}$, and $E^{*}$ are the values of the initial peak stress, plateau stress, and elastic modulus at 25 °C obtained from Equation (6). The parameters *T* and $\dot{\varepsilon }$ are the temperature and the strain rate. The terms $D_{i}(T)$, $D_{p}(T)$, and $D_{e}(T)$ are temperature damage functions. $M_{i}(\dot{\varepsilon })$, $M_{p}(\dot{\varepsilon })$, and $M_{e}(\dot{\varepsilon })$ are the strain rate functions [40,41,42] determined by the following equations:

(9) $$\left\{\begin{array}{c} M_{i}\left(\dot{\varepsilon }\right)={\left(\dot{\varepsilon }/\dot{\varepsilon_{0}}\right)}^{n_{i}}\\ M_{p}\left(\dot{\varepsilon }\right)={\left(\dot{\varepsilon }/\dot{\varepsilon_{0}}\right)}^{n_{p}}\\ M_{e}\left(\dot{\varepsilon }\right)={\left(\dot{\varepsilon }/\dot{\varepsilon_{0}}\right)}^{n_{e}} \end{array},\right.$$

where ${\dot{\varepsilon }}_{0}$ is the reference strain rate (500 s^−1^). The fitting parameters, *n*_i_, *n*_p_, and *n*_e_, depend on the temperature. Here, we took the specimen with a density of 0.46 g/cm^3^ as an example. The values of *n*_i_, *n*_p_, and *n*_e_ at temperatures of 25 °C, 200 °C, 400 °C, and 600 °C were calculated using the least-squares method, as shown in Table 6.

**Figure 17:**
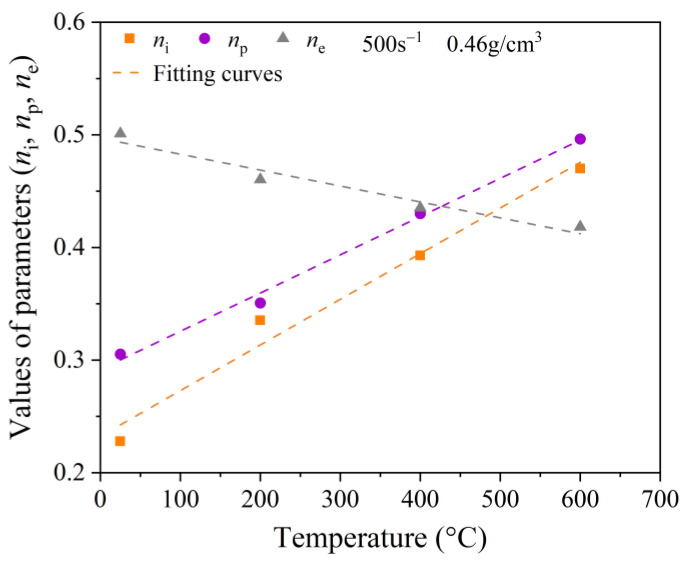
Fitted curves of Equation (8).

As shown in Figure 17, by fitting the data in Table 6, the relationship between *n*_i_, *n*_p_, and *n*_e_ and the temperature can be expressed as follows:

(10) $$\left\{\begin{array}{c} n_{i}=0.01T/T_{0}+0.23\\ n_{p}=0.0075T/T_{0}+0.29\\ n_{e}=0.0035T/T_{0}+0.5 \end{array}\right.$$

where *T*_0_ is the reference temperature of the specimen, which was taken as 25 °C.

Figure 11a–c show that the initial peak stress changes approximately linearly with the temperature. To describe this law, the temperature damage function for the initial peak stress at the strain rate of 500 s^−1^ can be written as follows:

(11) $$D_{i}\left(T\right)=-0.02T/T_{0}-1.02.$$

Through a detailed analysis of the results depicted in Figure 12a–c and Figure 13a–c, the temperature damage functions for the plateau stress and elastic modulus are established as follows:

(12) $$D_{p}\left(T\right)=\exp\left\{-\alpha_{p}\left[{\left(T/T_{0}\right)}^{2}-1\right]/\left[\beta_{p}+{\left(T/T_{0}\right)}^{2}\right]\right\},$$

(13) $$D_{e}\left(T\right)=\exp\left\{-\alpha_{e}\left[{\left(T/T_{0}\right)}^{3}-1\right]/\left[\beta_{e}+{\left(T/T_{0}\right)}^{3}\right]\right\},$$

where $\alpha_{p}$, $\beta_{P}$, $\alpha_{e}$, and $\beta_{e}$ are the fitting parameters for the proposed temperature damage functions, and their values are summarized in Table 7.

**Figure 18:**
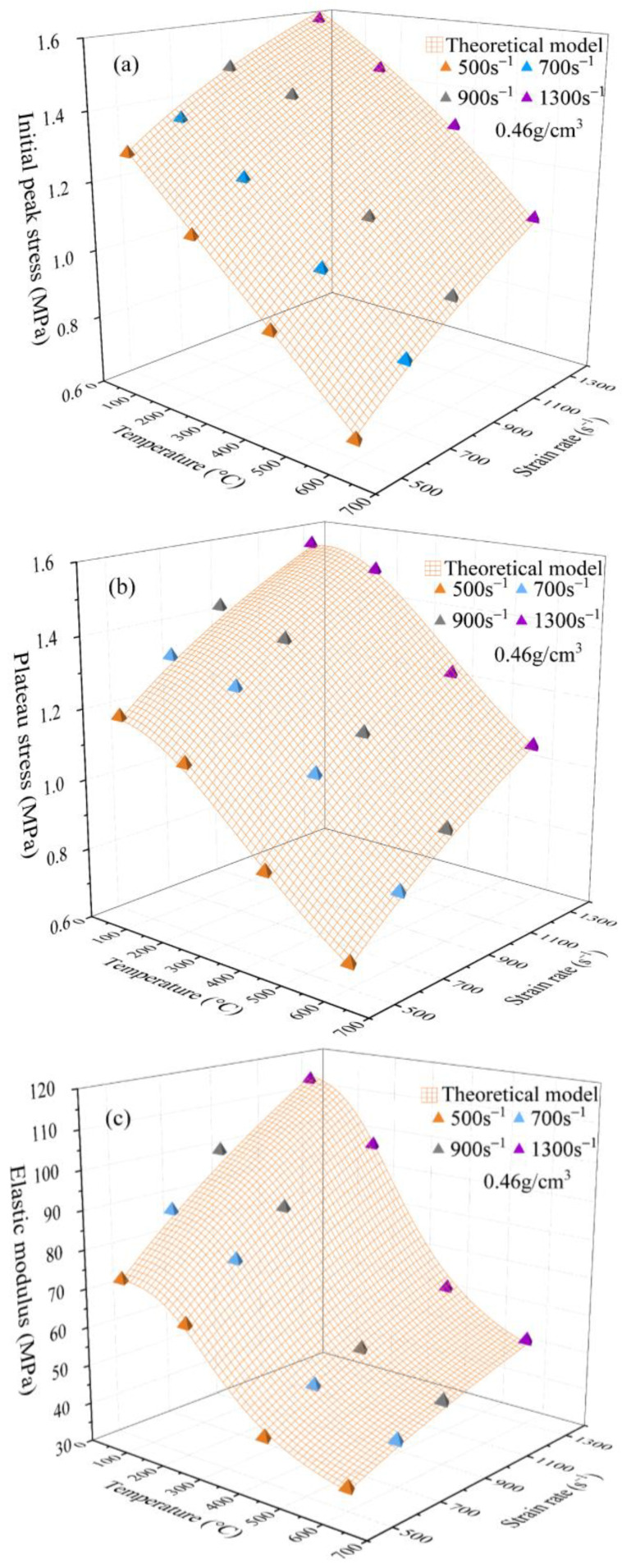
Three-dimensional plots of initial peak stress, plateau stress, and elasticity modulus versus strain rate and temperature. (**a**) Initial peak stress; (**b**) plateau stress; and (**c**) elastic modulus.

The predicted results acquired from the above model are shown in Figure 18, which shows a reasonable agreement with the experimental results. Therefore, this model is appropriate for describing the mechanical characteristics of the PPFRFC in the specified temperature and strain rate ranges.

## 4. Conclusions

In this paper, the mechanical properties of PPFRFC over a wide range of strain rates (500~1300 s^−1^) and temperatures (25~600 °C) are discussed. To conduct dynamic tests on the specimen at high temperatures, a modified SHPB technique was developed. A nylon bar was used to increase the duration of the incident wave, and a pair of mica plates were mounted at the bar–specimen interface for thermal insulation. The principal findings are succinctly summarized as follows:

(1) By mounting mica plates, the nylon loading bar can be effectively protected during high-temperature dynamic testing. Due to the close proximity of acoustic impedance between the mica plate and nylon, the influence of a mica plate with a thickness of 5 mm on wave propagation can be disregarded. This modified SHPB apparatus enables the possibility of conducting dynamic large-deformation tests on foam materials at high temperatures, which is helpful in investigating the high-temperature dynamic mechanical properties of the foam materials.

(2) The initial peak stress, plateau stress, and elastic modulus of the PPFRFC increase with increasing density. This density sensitivity decreases as the temperature increases, while it increases as the strain rate increases. However, the densification strain has a negative density sensitivity.

(3) The initial peak stress and plateau stress of the PPFRFC are sensitive to the strain rate, and their strain rate sensitivity is negatively dependent on the density and positively dependent on the temperature. In contrast, the strain rate sensitivity of the elastic modulus is positively dependent on the density and negatively dependent on the temperature. The densification strain of PPFRFC is affected by both the strain rate and temperature. It exhibits a negative strain rate dependence and a positive temperature dependence.

(4) A prediction model considering the strain rate and temperature effects is proposed. The predicted results show that the model can reliably reflect the dynamic response of PPFRFC at high temperatures. This model provides a reference for the safe utilization and design of PPFRFC under high-temperature and high-strain-rate conditions.

(5) The incorporation of PP fibers into PPFRFC specimens results in the marked enhancement of the crack resistance of the pore walls, leading to reduced fragmentation upon exposure to impact loading. When the temperature exceeds 200 °C, the specimen will produce more fragments due to the melting of the PP fibers. Additionally, the number and size of fragments are determined by the strain rate. Given these findings, the adoption of a high-temperature-resistant fiber as a reinforcement material may be beneficial in improving the dynamic mechanical properties of foam concrete at elevated temperatures. This factor should be taken into consideration in future research.

## Figures and Tables

**Table 1:** Mix proportions for specimens with densities of 0.27 g/cm^3^, 0.38 g/cm^3^, and 0.46 g/cm^3^.

Target Density (g/cm^3^)	Component (g/cm^3^)					Water-to-Cement Ratio
	Cement	Water	Foaming Agent	Fly Ash	PP Fiber	
0.27	0.17	0.17	0.0018	0.037	0.059	1
0.38	0.28	0.224	0.0016	0.061	0.084	0.8
0.46	0.33	0.231	0.0015	0.074	0.092	0.7

**Table 2:** Values of *σ*_i_, *σ*_p_, and *E* for specimens with a density of 0.27 g/cm^3^ at different strain rates and temperatures.

Temperature (°C)	Strain Rate (s^−1^)			Strain Rate (s^−1^)			Strain Rate (s^−1^)			Strain Rate (s^−1^)		
	500			700			900			1300		
	*σ*_i_ (MPa)	*σ*_p_ (MPa)	*E* (MPa)	*σ*_i_ (MPa)	*σ*_p_ (MPa)	*E* (MPa)	*σ*_i_ (MPa)	*σ*_p_ (MPa)	*E* (MPa)	*σ*_i_ (MPa)	*σ*_p_ (MPa)	*E* (MPa)
25	0.21	0.2	25.11	0.24	0.24	27.1	0.28	0.27	30.71	0.37	0.31	37.26
200	0.17	0.18	19.67	0.22	0.21	20.66	0.24	0.24	23	0.3	0.3	29.05
400	0.13	0.13	10.95	0.16	0.16	11.94	0.18	0.19	13.55	0.22	0.23	15.83
600	0.09	0.09	7.94	0.11	0.11	9	0.12	0.14	10.2	0.16	0.16	11.5

**Table 3:** Values of *σ*_i_, *σ*_p_, and *E* for specimens with a density of 0.38 g/cm^3^ at different strain rates and temperatures.

Temperature (°C)	Strain Rate (s^−1^)			Strain Rate (s^−1^)			Strain Rate (s^−1^)			Strain Rate (s^−1^)		
	500			700			900			1300		
	*σ*_i_ (MPa)	*σ*_p_ (MPa)	*E* (MPa)	*σ*_i_ (MPa)	*σ*_p_ (MPa)	*E* (MPa)	*σ*_i_ (MPa)	*σ*_p_ (MPa)	*E* (MPa)	*σ*_i_ (MPa)	*σ*_p_ (MPa)	*E* (MPa)
25	0.75	0.71	45.5	0.87	0.84	55.4	0.93	0.91	64	1.14	1.04	69.5
200	0.64	0.63	38.6	0.67	0.68	46.6	0.83	0.83	53.35	1.02	1	58
400	0.49	0.48	25.8	0.54	0.61	31.2	0.66	0.67	35.6	0.78	0.82	37.3
600	0.37	0.38	20	0.43	0.51	25.1	0.5	0.57	27.9	0.6	0.69	29

**Table 4:** Values of *σ*_i_, *σ*_p_, and *E* for specimens with a density of 0.46 g/cm^3^ at different strain rates and temperatures.

Temperature (°C)	Strain Rate (s^−1^)			Strain Rate (s^−1^)			Strain Rate (s^−1^)			Strain Rate (s^−1^)		
	500			700			900			1300		
	*σ*_i_ (MPa)	*σ*_p_ (MPa)	*E* (MPa)	*σ*_i_ (MPa)	*σ*_p_ (MPa)	*E* (MPa)	*σ*_i_ (MPa)	*σ*_p_ (MPa)	*E* (MPa)	*σ*_i_ (MPa)	*σ*_p_ (MPa)	*E* (MPa)
25	1.27	1.16	71	1.34	1.3	85.5	1.47	1.41	98.3	1.58	1.55	112.7
200	1.08	1.08	63.9	1.2	1.25	76.4	1.41	1.35	86.5	1.44	1.49	97.3
400	0.87	0.84	41.4	0.99	1.06	49.4	1.1	1.13	54.13	1.3	1.23	62
600	0.66	0.67	35.8	0.81	0.8	41.8	0.93	0.92	46.5	1.06	1.06	52.84

**Table 5:** Values of the fitting parameters *A*_i_, *A*_p_, *A*_e_, *m*_i_, *m*_p_, and *m*_e_ at different strain rates and temperatures.

Strain Rate (s^−1^)	Temperature (°C)	*A*_i_ (MPa)	*m*_i_	*A*_p_ (MPa)	*m*_p_	*A*_e_ (MPa)	*m*_e_
500	25	0.25	3.08	0.24	2.97	23.66	2.04
	200	0.21	3.09	0.21	3.1	17.75	2.38
	400	0.15	3.32	0.16	3.16	10.6	2.55
	600	0.11	3.37	0.12	3.32	7.32	3
1300	25	0.46	2.37	0.39	2.64	38	2.02
	200	0.39	2.51	0.37	2.64	30	2.2
	400	0.26	3.01	0.3	2.91	19.3	2.21
	600	0.18	3.3	0.23	2.93	14.59	2.44

**Table 6:** Parameter values for the proposed strain rate functions.

Temperature (°C)	*n*_i_	*n*_p_	*n*_e_
25	0.23	0.31	0.50
200	0.34	0.35	0.46
400	0.39	0.43	0.44
600	0.47	0.5	0.42

**Table 7:** Parameter values for the proposed temperature damage functions.

*α*_p_	*α*_e_	*β*_p_	*β*_e_
1.5	0.86	999	2886

## Data Availability

The data presented in this study are available on request from the corresponding author.
